# Advances in Hybrid Composites for Photocatalytic Applications: A Review

**DOI:** 10.3390/molecules27206828

**Published:** 2022-10-12

**Authors:** Stefania Porcu, Francesco Secci, Pier Carlo Ricci

**Affiliations:** 1 Department of Physics, University of Cagliari, S.P. No. 8 Km 0.700, 09042 Monserrato, Italy; 2 Department of Chemical and Geological Science, University of Cagliari, S.P. No. 8 Km 0.700, 09042 Monserrato, Italy

**Keywords:** photocatalysis, semiconductor photocatalysts, hybrid systems, organic/inorganic heterostructures

## Abstract

Heterogeneous photocatalysts have garnered extensive attention as a sustainable way for environmental remediation and energy storage process. Water splitting, solar energy conversion, and pollutant degradation are examples of nowadays applications where semiconductor-based photocatalysts represent a potentially disruptive technology. The exploitation of solar radiation for photocatalysis could generate a strong impact by decreasing the energy demand and simultaneously mitigating the impact of anthropogenic pollutants. However, most of the actual photocatalysts work only on energy radiation in the Near-UV region (<400 nm), and the studies and development of new photocatalysts with high efficiency in the visible range of the spectrum are required. In this regard, hybrid organic/inorganic photocatalysts have emerged as highly potential materials to drastically improve visible photocatalytic efficiency. In this review, we will analyze the state-of-art and the developments of hybrid photocatalysts for energy storage and energy conversion process as well as their application in pollutant degradation and water treatments.

## 1. Introduction: Importance and Limits

**Figure 1:**
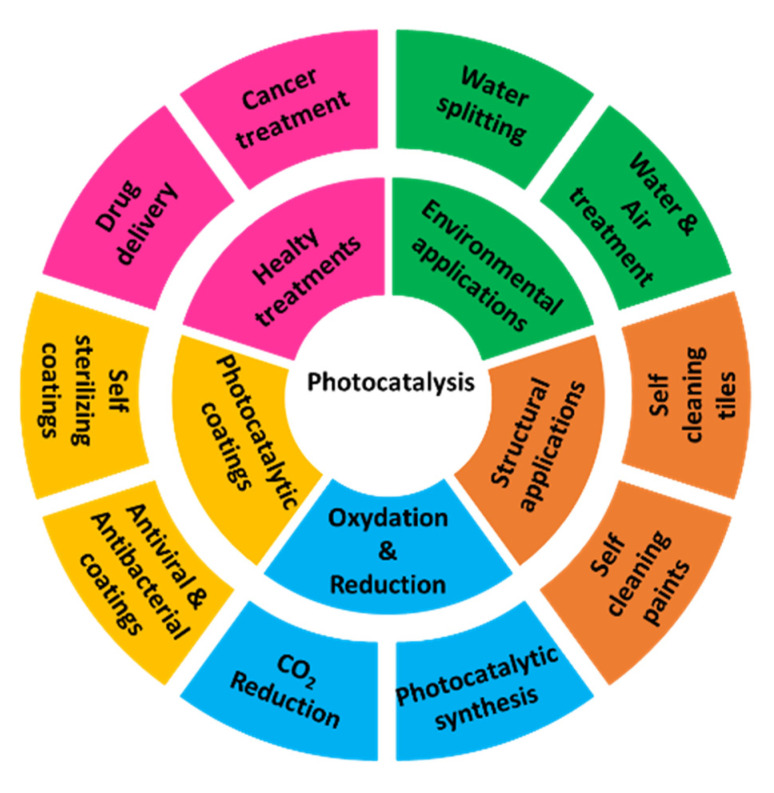
Application fields of photocatalysis.

The research and the development of new solutions that strengthen industrial and technological progress, guaranteeing sustainable impacts, is greatly boosting the effort of the scientific community. Modern household combustion devices, motor vehicles, and industrial facilities are common sources of air and water pollution. Particulate matter, nitrogen oxide, sulfur dioxide, volatile organic compounds, dioxins, and polycyclic aromatic hydrocarbons are considered pollutants that are greatly harmful to humans. Outdoor and indoor air pollution cause respiratory and other diseases and are important sources of morbidity and mortality. Policies to reduce air and water pollution represent a winning strategy for both climate and health, lowering the burden of disease attributable to air pollution, as well as contributing to the near- and long-term mitigation of climate change. An interesting review of the environmental and health impact of air pollution is reported by Manisaldis et al., [1] analyzing the sources of environmental pollution in relation to public health and environmental effects. Boosting education, training, public awareness, and public participation are some of the relevant actions indicated in the Paris Agreement of 2015, issued by the UNFCCC (United Nations Climate Change Committee) for maximizing the opportunities to achieve a sustainable impact by the anthropogenic actions, mitigating the climate change and environmental pollution [2]. The raising of environmental technologies can greatly support political/social actions and virtuous daily behaviors. Some examples can be sketched, such as the use of renewable energy obtained by sunlight, wind, rain, waves, and geothermal heat, the definition of smart mass transportation, and highly efficient electric vehicles. However, several factors should be considered in the definition and the use of “green” technologies, starting from the materials and their complete cycle of life, the efficiency, the environmental impact, and the up-grading possibilities. On these bases, photocatalysis is strongly indicated as a promising technology having a fundamental role in applications such as fuel cells, solar conversion, healthy treatments, and environmental radiation (Figure 1). If water splitting has been intensively studied as a possible approach to achieve the effective conversion of solar into chemical energy, photocatalysis is also utilized to decrease the number of organic compounds in the air, water, or soil, representing a high environmental risk for health. 

Photocatalysis has been applied to degrade several types of organic waste, such as dyes [3,4], phenolic compounds [5,6], organohalides, and petroleum hydrocarbons [7], but also for the removal of heavy metals such as Chromium, Lead, or Mercury among the others [8,9]. The photocatalytic techniques have been applied even to the degradation of pharmaceutical compounds (PC) that represent a new and emerging source of waste in water and soils [10]. Antibiotics and their by-products have high toxicity, good stability, and great potential to interfere with the environment and ecological environment. Many antibiotics are discharged into the water environment through sewage and animal feces, causing severe water and soil environmental problems [11]. Anti-inflammatory drugs have a high polarity and strong hydrophilicity, but the absorption coefficient in the soil is low, so they are easy to survive underground, surface, and even drinking water resources, causing great pollution to water resources.

**Figure 2:**
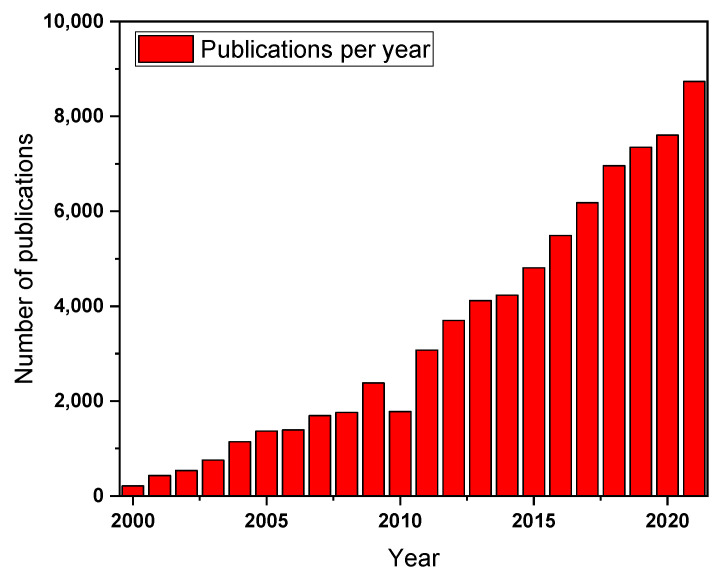
Representative scheme of the publication rate of papers regarding photocatalysis.

The term “photocatalysis” and the base of the effect are already known from the end of the 19th century when Giacomo Ciamician defined the term photocatalysis for the first time. Successively, it this worth mentioning that the first results on photocatalytic water splitting using a TiO_2_ electrode were reported by Fujishima and Honda in 1972 [12]. After that, the research on this field strongly increased, and in the last 20 years, the number of publications on “photocatalysis” has constantly grown up to 8737 publications in 2021 (Figure 2).

Moreover, the “old” knowledge, the research in this field, and, mostly, the development of new materials with higher efficiency is a mandatory step to overcome the actual technical limits of the processes. Most photocatalysts can utilize the sizeable visible component of the solar spectrum very partially, working on higher energy photons (UV range) that count for only 5% of the solar emission.

Recently different strategies and materials have been utilized to overcome this issue: lowering the band gap of the materials, new energetic levels by doping process, and heterostructures with increased charge separation are just a few examples that we better discuss in the review. On the other hand, the achievement, or at least the improvement in this direction, has a strong potential impact on our daily life and, mostly, on the environmental aspect for a long-time vision of sustainable progress. 

Sunlight is a free, perpetual, and clean energy source. If we consider that photocatalysis is a light-induced chemical pathway able to convert organic material into energy, the benefits of this process appear immediate. However, even if the energy produced from the sun is renewable and gives us a plethora of advantages, the materials needed to convert it into practical use should be carefully chosen to guarantee an environmentally friendly process. They should be free from potential impact connected to the extraction of the raw materials and precursors, the use of solvents in the synthesis procedure, and the energy required in the whole life cycle of the photocatalysts should be carefully checked [13,14].

Among the wide choice in terms of possible materials, photocatalysts can be classified into two main categories: semiconductors (organic or inorganic) and metal nanoparticles. Transition metals such as gold, copper, and silver are characterized by localized surface plasmon resonance (LSPR), while semiconducting materials such as TiO_2_, ZnO, SnO_2,_ MoS_2_, Bi_2_MoO_6_ [15,16,17,18,19,20] assure complete mineralization, no waste disposal problem, low cost and necessity of mild temperature and pressure conditions. Organic materials show mechanical and chemical properties that radically differentiate them from inorganic ones. The absence of metals, the high possibilities to tune the synthesis technique, and the high varieties of optical and structural character of the organic photocatalyst, justify the high interest in this class of materials. Within the organic chemistry panorama, polymers can guarantee very high versatility in terms of structural and optical response with high potential in several applications, but their efficiency is generally hampered by the low thermal and short-time structural stability of the compounds. As already mentioned, different thousands of articles and studies are constantly published, and the interest of the scientific community is strongly active in this field. On these bases, it is important to summarize time by time, the state-of-the-art progress in the design of a variety of semiconductor composite photocatalysts for energy and environmental applications. In recent years different review articles were focalized on photocatalysis, from base properties [21,22,23,24] to materials [25,26,27,28] and to applications. 

Inside these very broad categories, several aspects have been considered. If Humayun et al. [29] pointed out the synthesis procedures of composite photocatalysts and the use of microwave is underlined, other works focalize on a specific class of materials: MOF [25], metal halide perovskite [26], graphene [28], and carbon nitride [30].

The use of the photocatalytic process in water treatments [31], for the removal of Volatile Organic compounds (VOC) [32], and the abatement of plastics [33] are just a few recent examples, such as its use for the degradation of persistent herbicides [34] or, more in general, for the development of sustainable agriculture [35].

In this review, we will briefly analyze the state art in inorganic and organic semiconductor photocatalysts, mainly focalizing our discussion on the method to obtain efficient processes using visible light. We will underline the efforts of the scientific community to build efficient heterostructures, and we will analyze the new solutions based on hybrid systems, organic-inorganic, that efficiently work with a relative portion of the solar spectrum. 

## 2. Basic Principles of Photocatalysis

**Figure 3:**
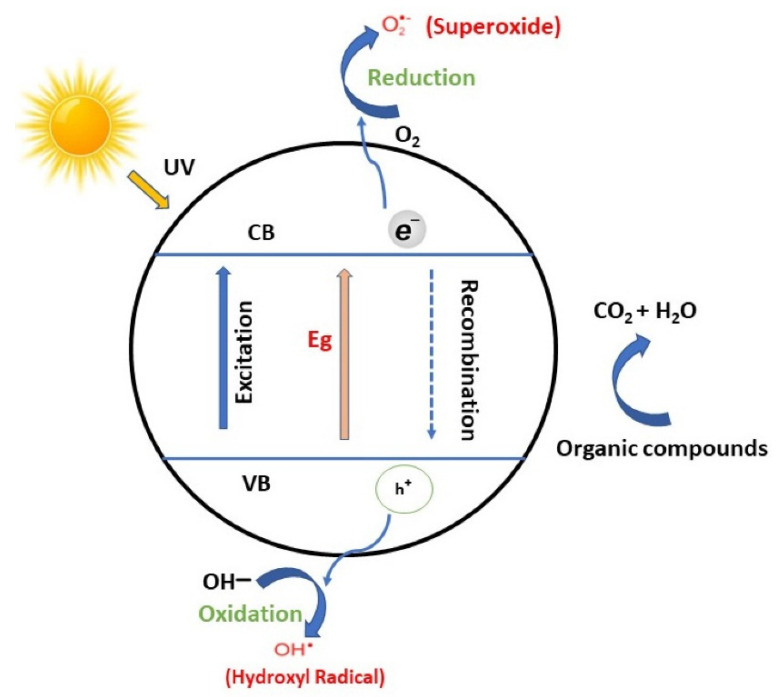
Basic scheme of semiconductor−mediated photocatalytic process [38].

A Photocatalytic process occurs when the light interacts with the surface of semiconductor materials, the so-called photocatalysts, allowing the photo redox process. During this process, there must be at least two simultaneous reactions occurring, oxidation from photogenerated holes and reduction from photogenerated electrons (Figure 3) [36,37,38].

The process can be divided into four fundamental steps: (I) light absorption to generate electron-hole pairs; (II) separation of excited charges; (III) transfer of electrons and holes to the surface of photocatalysts; (IV) utilization of charges on the surface for redox reactions [39]. The holes left in the valence band have high oxidizing power and, reacting with water, generate hydroxyl radicals responsible for pollutant degradation. The electrons in the conduction band, via reaction with dissolved oxygen species, form superoxide ions that promote the reduction process [36,40].

In the oxidation process, the positive holes in the valence band allow oxidizing of the water at the surface of the catalyst, forming hydroxyl radicals (OH^.^) with a high oxidative decomposing power. These hydroxyl radicals react with the organic pollutant that, at the end of the process, is decomposed into carbon dioxide and water. As well, the organic compounds could react directly with the positive holes created in the valence band, activating an oxidative decomposition.

The reduction process of the oxygen contained in the air is an alternative reaction to hydrogen generation since oxygen is an easily reducible substance. The electrons in the conduction band, reacting with the dissolved oxygen species, form superoxide anions that, after being attached to the intermediate products obtained from the oxidative reaction, form peroxide or hydrogen peroxide and then water as the final product.

The photocatalytic activity of a material depends on several parameters: morphology, size, surface area, reaction temperature, pH, light intensity, amount of catalyst, and concentration of wastewater dependent. The structure of the catalyst plays a fundamental role in the photocatalytic activity: the stability of the catalyst, the position of the conduction band, the degree of hydroxylation and the absorption power, and the size of the particles. In general, the smaller the size of the catalyst nanoparticles, the higher the photocatalytic activity. Cerruto et al. systematically correlated the photocatalytic activity to the average sizes and size distributions of TiO_2_ nanocrystals [41], while more recently, the study was extended to the hydrogen produced by Al-doped SrTiO_3_ photocatalysts [42] to the visible photocatalytic activity in β-Bi_2_O_3_ [42,43], and the reduction of CO_2_ in Cu/Cu_2_O nanocrystals [44]. The surface properties significantly influence the efficiency of the catalyst because, during the photocatalytic process, oxidation and reduction reactions take place on the surface of the catalyst [45,46].

On this topic, He et al. found that the differences in the photocatalytic activity of different shaped samples of ZnO are not caused by the changes in specific surface area, oxygen defects, or particle size, but it was attributed to the pore size. The pore size determines the adsorption efficiency of organic pollutants and dissolved oxygen, and good adsorption performance is the main prerequisite for efficient catalytic degradation [47].

An increase in the temperature during the photocatalytic process induces a decrease in photocatalytic activity. Since the activation is due only to photonic interaction, the photocatalytic systems can operate at room temperature [48]. Even pH plays a key role in photocatalytic reactions because it is responsible for the surface charge properties of the photocatalyst. This could be explained in terms of electrostatic interactions between charged particles and contaminants, influencing the absorption and, consequently, the surface properties [49,50,51]. From multiple studies, it was also evaluated that the degradation rate is directly proportional to the catalyst concentration. An increase in the amount of the catalyst during the photocatalytic process is reflected in the highest number of active sites formed on the semiconductor surface and in an increasing number of OH^.^ and O_2_**^−^**^.^ radicals [52].

Another fundamental factor in estimating the degradation rate during a photocatalytic process is the type of pollutant and its concentration. Having different pollutants and different irradiation times to complete mineralization is needed [36,48,52,53].

## 3. Semiconductor Photocatalysts

**Figure 4:**
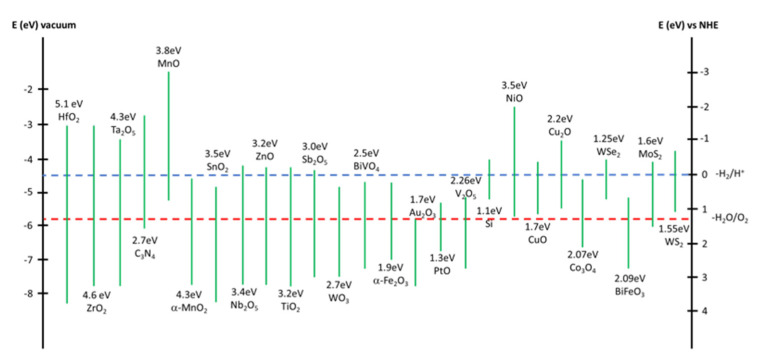
Semiconductor’s band−gaps and redox potentials.

Photocatalysts are defined as a class of materials that absorb light and can convert it into electrical charges driving the reduction and/or oxidation of species at the catalyst surface. During the last three decades, great attention has been focused on semiconductor metal oxides, selenides, and sulfides as photocatalysts thanks to their bandgap energy between 1.1 and 3.8 eV and their redox potential (Figure 4) [40]. The species with a higher reduction potential have a higher tendency to acquire electrons, while the species with a higher oxidation potential tend to lose them and to be oxidized.

Metal oxides are largely considered the most efficient and widely applied materials, while metal selenides and sulfides are poorly considered for their toxicity and poor stability [46,54]. In general, the photocatalytic mechanism in semiconductors can be sketched with the below-mentioned Equations (1)–(10).

*Semiconductor* + *Light*
*energy* → *Semiconductor*
*(e*_*cb*_^−^ + *h*_*vb*_^+^*)* (1)

*Dye* + *Semiconductor*
*(h*_*vb*_^+^*)* → *Oxidation process* (2)

*Semiconductor (h*_*vb*_^+^) + *H*_*2*_*O* → *Semiconductor* + *H*^+^ + *OH*^.^ (3)

*Semiconductor (h*_*vb*_^+^*)* + *OH*^−^ → *Semiconductor* + *OH*^.^ (4)

*Dye* + *Semiconductor (e*_*cb*_^−^*)* → *Reduction process* (5)

*Semiconductor (e*_*cb*_^−^) + *O*_*2*_ → *Semiconductor* + *O*_*2*_^.−^ (6)

*O*_*2*_^.−^ + *H*^+^ → *HO*_*2*_^.^ (7)

*HO*_*2*_^.^ + *HO*_*2*_^.^ → *H*_*2*_*O*_*2*_ + *O*_*2*_ (8)

*H*_*2*_*O*_*2*_ + *O*_*2*_^.−^ → *OH*^.^ + *OH*^−^ + *O*_*2*_ (9)

*Dye* + *OH*^.^ → *Degradation Products* (10)

Currently, the most attractive photocatalytic materials are n-type semiconductors such as TiO_2_, WO_3,_ and Fe_2_O_3_, because of their high chemical stability and a conduction band edge with a potential level that is negative enough to allow the proton reduction without additional electric bias [55,56,57,58].

As already stated, the first studies of TiO_2_ as a photocatalyst it is date back to 1972, and then in 1977, Bard applied it for the first time to purify water from CN^−^ ions [59].

To date, TiO_2_ is considered an n-type semiconductor with several advantages (high photoactivity, strong oxidizing capability, cost-effectiveness, low toxicity, and high stability) and represents one of the most widely studied and applied semiconductor photocatalysts. On the other hand, it still suffers, such as all the inorganic semiconductors, of high recombination rate and negligible light harvesting in the visible range.

**Figure 5:**
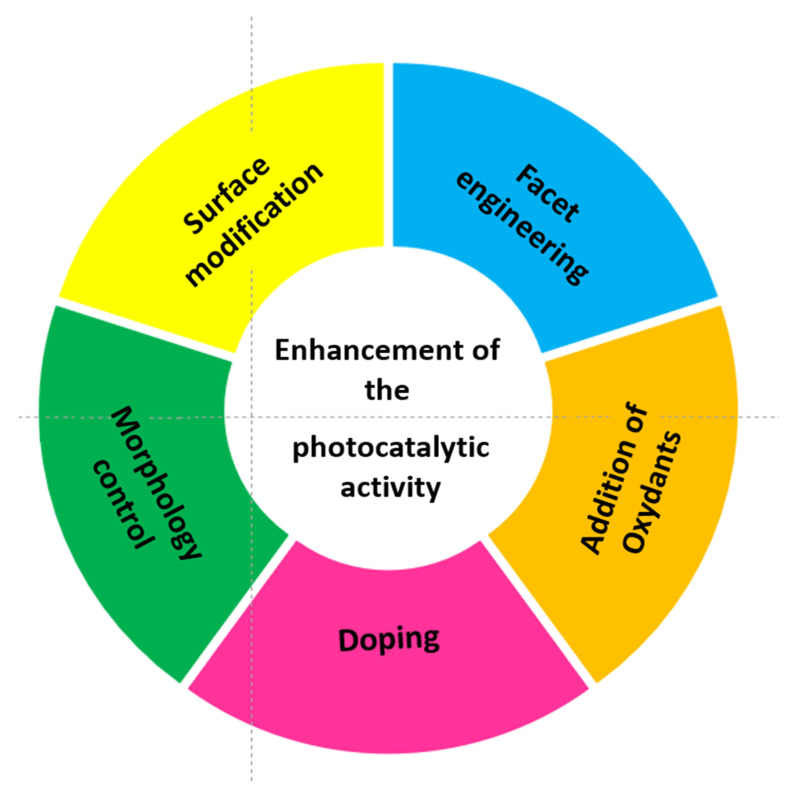
Strategies to enhance the photocatalytic activity.

In order to overcome the bottlenecks above, the research has been focused on improvements in solar spectral absorption and decreasing the recombination rate of these photocatalysts (Figure 5).

The regulation of the energy band structures is the best way to enhance the visible light photocatalytic performance, generally by doping, defects engineering, and surface modification.

Li et al. attribute the increased efficiency of Carbon doped TiO_2_ nanofiber under visible light to the presence of oxygen vacancies at the surface while the Ti terminals act as reaction sites, resulting in the accumulation of CO* intermediates to produce CH_4_ [60]. The role of C doping is further analyzed by Nor et al., By DFT calculations. They suggest that C-doped O sites are more stable in bulk than in the subsurface or on the surface, while C-doped Ti sites are more stable on the surface than in the bulk or subsurface. While the presence of C in the O sites introduces impurity states in the band gap that do not affect the band gap energy but induce the formation of electron traps, Carbon in Ti sites induces structural distortions with a reduction in the band gap energy. Both doping processes enhance light absorption in the visible and IR spectrum [61]. The role of lanthanides as doping elements and/or surface modification has been widely studied. The presence of Dy generates a ligand-to-metal charge transfer process in the visible region (500–600 nm), lowering the bandgap to ~2.24 eV [62]. Guetni et al. found that doping TiO_2_ with Nd allows the material to absorb radiation in the visible range by creating Nd-4f impurity states. The co-doping with Y further increases the Azo dye orange G degradation under a wavelength range of 300–800 nm due to the synergetic effect of intragap levels [63].

The increased visible-light photoactivity of metal-doped TiO_2_ by intragap levels in the semiconductor band gap requires photons with lower energy to produce carriers in the conduction band. It is well known that in N-doped TiO_2,_ the lowered band gap is due to the formation of N-2p state hybrids with O-2p states in the anatase phase [64]. Further, the improved trapping of electrons at oxygen vacancy sites can inhibit the electron-hole recombination resulting in enhanced photo-activity [65].

A different mechanism is related to the formation of Oxygen vacancies at the surfaces of the semiconductors (related to metallic doping) and, consequently, to F centers with different charges. The loss of an O atom in a metal oxide generates an electron pair trapped in VO (F center), while a positively charged F+ center is due to a single electron in the O vacancy. An unsaturated vacancy generates an F++ center [66]. The presence of charge defects strongly increases the surface reactivity and, as a consequence, the photocatalytic activity, too. 

Various metal nanoparticles such as Pt, Au, Pd, Ru, Rh, and Ag have been employed in implementing the semiconductor surface to achieve higher photocatalytic efficiency. High energy irradiation induces Fermi-level equilibration between the semiconductor (like TiO_2_) and the metal element through charge distribution [67,68], strongly increasing the electron transfer on the semiconductor/metal interfacial system and promoting efficient photocatalytic reactions. The presence of resonance factor related to the reduced dimension of the metal nanoparticle at the surface (mainly Au and Ag) can give act as an added mechanism.

A different approach consists of the use of carbon aerogels materials hybridized with TiO_2_; the main effect is related to the increase in the surface area of TiO_2_ due to the great porosity and highly developed hierarchical porous structures. Further, Carbon functionalization results in the formation of new intragap levels that red-shift the absorption in the visible range and, acting as a charge trap, limit the recombination rate [58]. However, there are still some issues to be solved, firstly, the efficiency in the photodegradation should be still increased, but mainly there are some issues related to the synthesis procedure that requires intensive energy consumption and the use of a considerable amount of organic precursor such as formaldehyde [69].

Localized surface plasmonic resonance (LSPR) can be seen as the collective free electron charge oscillation in the metallic nanoparticles optically consistent with the frequency of the incident light wave [70]. This phenomenon usually occurs in nanoparticles below 50 nm and creates an added optical excitation band in the visible region (480 for Ag, 520 for Au) [71].

Yukika et al. coupled small Pt nanospheres with large Au nanocubes on TiO_2_ to obtain a coupling resonance mode at about 600 nm. Further, the bimetallic coupling allowed charge separation and catalytic reaction at the Pt site and a light-harvesting antenna at the Au site, respectively [72].

It was observed that catalytic reduction of CO_2_ can be obtained with Au nanoparticles deposited TiO_2_ under full solar-spectrum irradiation, thanks to the visible contribution associated caused with the LSPR effect of Au NPs [70,73]. A further method to increase the photo-efficiency foresaw the synergic effect of the localized plasmonic features and the formation of metal chiral structures. Indeed, plasmonic materials with chiral configurations have not only emulated the optical properties of their molecular counterparts, but the chiroptical activity can achieve 10 orders of magnitude greater [74,75]. The great enhancement of the electric field, as well as the formation of hot injecting electrons in the semiconductor inside and around the plasmonic nanostructures, are the main player in the mechanism.

Organic semiconductors have recently gained large interest due to the earth’s abundance of their constituent elements, optical and structural properties, and in particular, their tunable energy levels. It is outside the scope of the present paper, but interesting examples of reviews on the use of organic semiconductors in photocatalytic applications are reported in Refs. [76,77].

**Figure 6:**
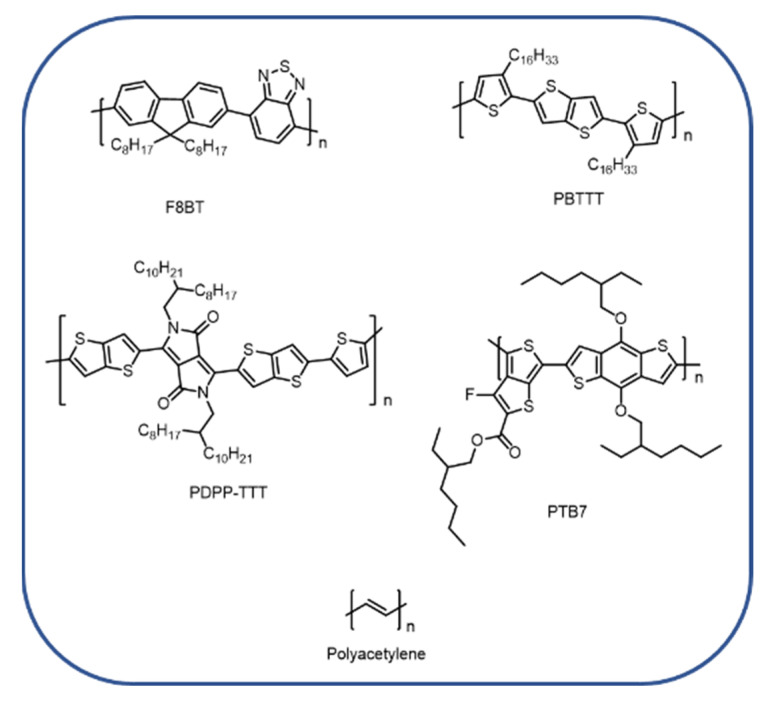
Chemical structures of polymeric semiconductors.

This class of materials can be divided into two categories: polymers and small molecule materials. Polymeric macro-molecules are constituted by the repetition of a fundamental unit (monomer) and are based on *sp2* hybridized carbon atoms, with the alternation of single and double bonds along their building block (Figure 6).

**Figure 7:**
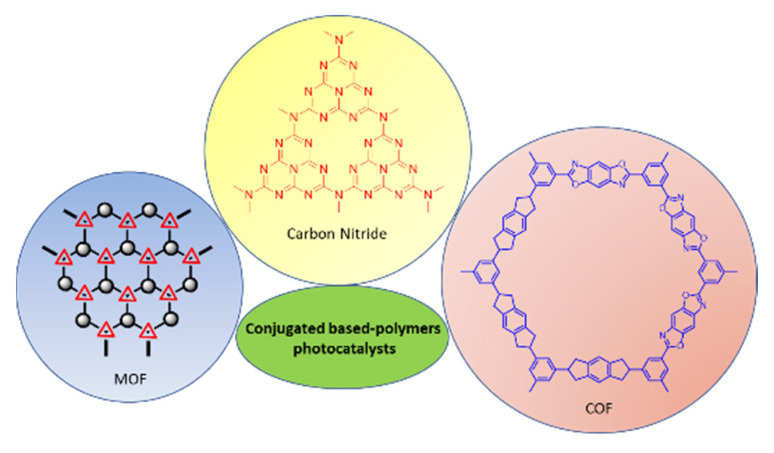
Examples of conjugated polymer photocatalysts.

Within the organic chemistry panorama, polymers can guarantee very high versatility in terms of structural and optical response with high potential in several applications [78,79,80], but their efficiency is hampered by the low thermal and short-time structural stability of the compounds (Figure 7).

Covalent organic frameworks (COFs) are a type of purely organic crystalline porous polymeric material with a very high structural tunability due to the high possibility of varying the various functional building blocks [81,82]. COF can be tuned to vary the absorption into the visible range up to infrared with relatively high efficiency. Further, the large specific surface area and regular and customizable micro- and mesopores can offer increased interaction between substrates and catalytic sites. Furthermore, via bottom-up synthesis, post-modification, doping, photosensitization, and hybridization with other photoactive compounds, the construction of the heterojunction structure, the electron-hole recombination can be greatly retarded, the charge transfer processes can be accelerated, and the charge transfer resistance can be greatly decreased [12]. Moreover, the considerably large specific surface area and numerous regular and customizable micro- and mesopores can offer favourable guest-host interaction between substrates and catalytic sites. The building blocks to produce the organic porous architectures are generally rigid and do not possess bulky side groups.

In this scenario, polymeric carbon nitrides have emerged as promising materials for being metal-free semiconductors and metal-free photocatalysts.

First attempts to prepare solid-state carbon nitride materials have been known in the literature since early 1830, and they have eluded rigorous analyses and characterizations for a long time because of their insolubility in water and organic solvents.

Kroke et al., using density functional theory methods (DFT), proposed a polymer based on two-dimensional tri-s-triazine units (heptazines) as the most thermodynamically stable structure of carbon nitride [83]. This polymer, because of its graphitic-like structure, was then called and studied graphitic carbon nitride (g-C_3_N_4_) and has attracted strong attention because of its tunable optoelectronic properties and because it is a metal-free semiconductor [84,85,86]. The chemical structure of graphitic carbon nitride has been deeply studied with different techniques, including solid-state nuclear magnetic resonance spectroscopy, Fourier-transform infrared spectroscopy, and x-ray diffractometry [87]. These studies confirmed that g-C_3_N_4_ is a layered material, in which van der Walls force holds the stacking layers (C-N bonds), and each layer is composed of tri-s-triazine units, bridged by tertiary amino groups with a high degree of condensation. It is thermally and chemically stable under ambient conditions, biocompatible, eco-friendly, and the graphite-like planar configuration, with a π conjugated system, thereby enabling the transport of charge carriers, and the moderate bandgap makes it workable in the visible part of the solar spectrum [88,89,90,91]. The lone pair electrons of nitrogen are responsible for the formation of the lone pair valence band and the band structure. The combination of the lone pair state and the p bonding stabilizes the lone pair position becoming the key to the electronic structure [92,93].

These appealing properties make g-C_3_N_4_ useful for different applications, and from 2000–2019, around 5100 publications have been published on g-C_3_N_4_ applications, including photocatalytic water splitting, CO_2_ reduction, environmental remediation, organic transformation, and white light-emitting diodes [19,94].

**Figure 8:**
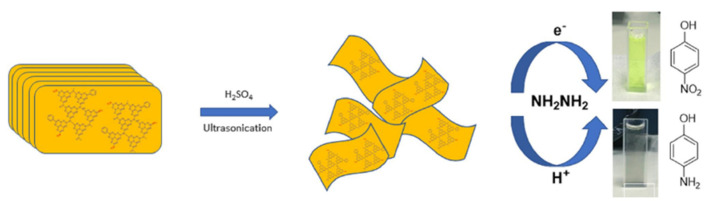
4−Nitrophenol reduction using protonated and exfoliated phenyl carbon nitride (pePhCN) as photocatalyst [97].

Despite these positive attributes, the efficiency of g-C_3_N_4_ as a standalone photocatalytic material is diminished by a low quantum efficiency and high recombination rate of photogenerated electron-hole pairs [95]. However, the modification of g-C_3_N_4_ could overcome these problems. Recent studies reported that by controlling the morphology and the surface properties of these catalysts, a significant enhancement in photo-reduction is observed. In particular, Porcu et al. studied a new polymer of the family of carbon nitrides with enhanced absorption in the visible part of the solar spectrum [96]. They showed how the protonation method could improve the solubility, dispersibility, and electronic structure, and the exfoliation method of 2D structures, through the breaking of Van der Waals forces, can improve light absorption (Figure 8) [97].

## 4. Heterostructures 

One of the most effective strategies for improving the photocatalytic efficiency of semiconductor materials is the design of heterojunction systems [98,99,100]. A general rational design and fabrication of heterojunction photocatalysts, such as the semiconductor–semiconductor heterojunction, the semiconductor–metal heterojunction, the semiconductor–carbon heterojunction, and the multicomponent heterojunction can be found in the review of Wang et al. [101].

**Figure 9:**
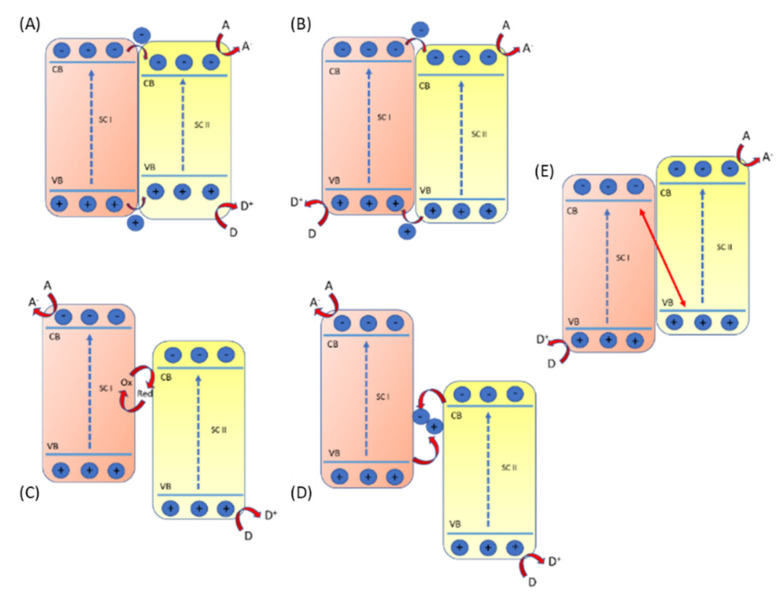
Heterostructures. (**A**) type−I (**B**) type−II (**C**) Z−scheme with redox mediator (**D**) all solid Z−scheme (**E**) S−scheme.

Heterojunction photocatalysts are obtained by coupling two semiconductor photocatalysts and taking advantage of their relative band structure [102,103]. The coupling of the two systems has been classified into Type I, Type II, Z, and S-scheme photocatalysts. (Figure 9) [29,98].

In type I, the heterostructure is formed by two semiconductors, and the photoinduced electron and holes are transferred from one semiconductor to the other, but due to the electron-holes accumulation in one semiconductor, the redox ability is relatively low. Type-II heterojunction has been widely studied and applied, starting in the 1980s [104]. It is based on the simultaneous formation, upon a proper excitation wavelength, of electrons and holes in both photocatalysts. Photogenerated electrons were transferred from one photocatalyst to the other, while photogenerated holes were transferred in the opposite direction. Thus, the photogenerated carriers are spatially separated, and electrons and holes accumulate on different photocatalysts, one for the reduction reactions and the other for oxidation reactions, respectively. 

Despite the interesting process and the wide application and studies applied in this scheme [105], the type II scheme presents a low redox ability. Actually, from a dynamic perspective, the repulsion from the existing carrier (electron and carrier) already accumulated in one photocatalyst hampered the transfer process, weakening the overall redox ability of the heterojunction [106,107].

The Z-scheme composites show a high redox potential and very efficient charge carrier separation. The photoinduced holes of semiconductor I recombine with the photoinduced electrons of semiconductor II leaving the photoinduced electrons of semiconductor I and the photogenerated holes of semiconductor II free to be active in the reduction and oxidation process, respectively [108]. These kinds of heterostructures find application in many fields, such as CO_2_ conversion, water splitting, and pollutant degradation [56,109,110].

The concept of the “direct Z-scheme” has been developed gradually. One of the first successful applications was the dye-sensitized TiO_2_ by Gratzel [110], where a tandem cell was obtained by coupling nanocrystalline WO_3_ or Fe_2_O_3_ with dye-sensitized TiO_2_. In this case, holes in the valence band of WO_3_ or Fe_2_O_3_ generated by UV radiation are involved in a water oxidation reaction, while the electrons, transferred to dye-sensitized TiO_2,_ will generate hydrogen in the water reduction reaction. Further, Balayeva et al. developed a surface-grafted WO_3_/TiO_2_ hybrid system with high photoactivity under visible light irradiation for indoor air purification [111]. Another Z-scheme application is ZnO-CdS Z-scheme heterojunction for improved H_2_ evolution performance and, more recently, Cu_2_O-Pt/SiC/IrOx heterostructure for CO_2_ reduction and O_2_ evolution [112]. Indeed, the second generation of Z-scheme photocatalysts considers the use of a mediator between the two materials. The close contact between the photocatalysts and the mediator allows the faster recombination between the photogenerated carriers (electrons from PS II with the photogenerated holes from PS I) due to a lower contact resistance interface [113].

Recently a new scheme has been proposed, the so-called S-Scheme. An S-scheme is like a type-II heterojunction but with a different charge-transfer path. In a typical type-II heterojunction, photogenerated electrons and holes are accumulated on the conduction band and valence band of the two semiconductors resulting in weak redox ability. In an S-scheme heterojunction, the photogenerated electrons and holes are stored in the conduction band of the reduction photocatalysts and the valence band of the Oxidative photocatalyst, respectively, while the other photogenerated charge carriers of the two semiconductors are recombined together [98].

Jang et al. [114] report superior performances for H_2_O_2_ production in a heterojunction between ZnO and WO_3_. The formation of the S-scheme heterojunction (ZnO and WO_3_) generates electrons on the conduction band of ZnO and holes on the valence band of WO_3_ that are efficiently spatially separated. Further, CdS/TiO_2_ heterostructures with a working s-scheme are reported by Wang et al. Due to the higher different working function, electrons from CdS can transfer to TiO_2_, forming the internal electric field directing from CdS to TiO_2_. Under illumination, the photogenerated holes in the valence band of CdS recombine with the photogenerated electrons in the conduction band of TiO_2_ driven, leaving the photogenerated carriers (electron in the conduction band of CdS and photogenerated holes in the valence band of TiO_2_) free to act to the CO_2_ reduction and water oxidation reactions, respectively [115].

The S-scheme has been highly utilized in the hybrid organic-inorganic heterostructures, and it will be recalled in the following paragraph.

## 5. Organic/Inorganic Heterostructures

**Figure 10:**
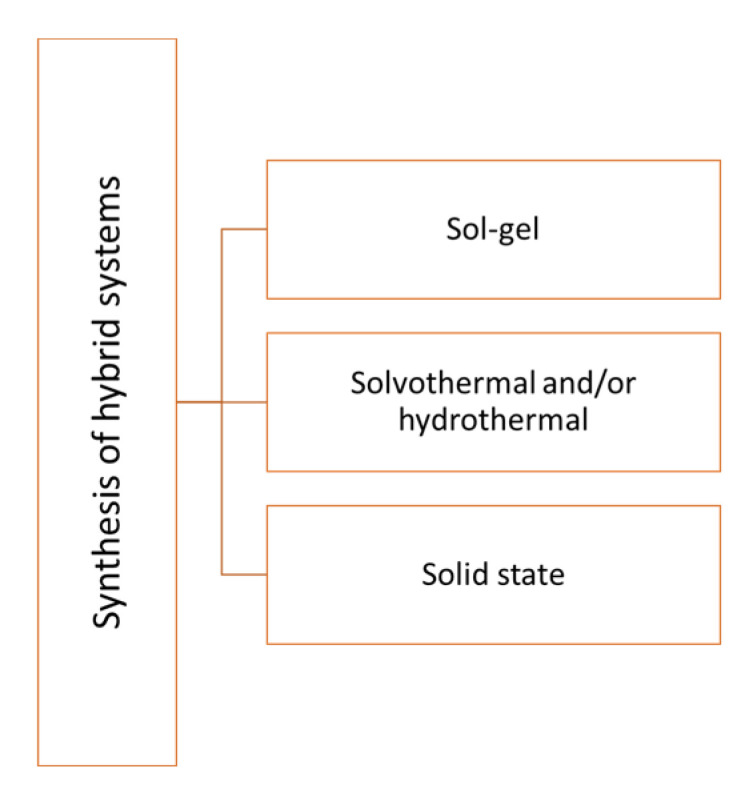
Most representative synthetic methods to obtain hybrid systems.

The development of new materials with higher activity and versatile properties for photocatalytic applications is strongly required. The formation of “hybrid materials”, by coupling an inorganic material (often already in the list of the classical photocatalysts) with new organic structures (often represented by stable polymers) permits to obtain a new class of materials with superior and tunable properties (Figure 10).

Depending on the type of bonding between the two materials, different effects and classifications can be defined. In the first case, the stability of the structure is provided by the presence of physical interactions, such as hydrogen bonding, van der Waals force, or ionic interactions. Proper functional groups at the surface are necessary to stabilize the interaction and stabilize the structure. The second type is defined by stronger interactions generated by chemical bonding, and in general, it defines very stable hybrid materials and a fast charge transfer process.

The organic part in the hybrid system can either be molecular catalysts or organic dyes, which can act as photocatalysts or sensitizers. In this latter case, they will not act as a photocatalyst but support the new system with their properties. The general mechanism is relatively easy: the sensitizer, chemically adsorbed on the semiconductor surface, absorbs the photon with a suitable wavelength passing to an excited state, then the photoexcited carriers are injected into the excited inorganic band (following one of the above descript mechanism, such as Z-scheme and/or S-Scheme). The main scope of the sensitizer is to overcome the physical limitations of the inorganic semiconductors, which generally can absorb only the UV component of the electromagnetic spectrum. Different systems were proposed, where both the inorganic part as well the organic units have been varied. Metal oxides still represent the most common choice, while much more variety can be found in the organic choice. 

The π-conjugated systems are promising materials as sensitizers in hybrid systems. Suárez-Méndez et al. [116] report the increased photocatalytic performance of protonated thiophene-based oligomers (OTn+) as sensitizers in an OTn/TiO_2_ system, while Zhong et al. show high H_2_ production activity using excitation wavelengths greater than 420 nm in a porous organic-inorganic hybrid system formed by Calix [4] arene dye as sensitizer and titanium dioxide as acceptor units [117].

Coumarins represent a further interesting material for the high absorption cross-section and guarantee an efficient and fast charge transfer to TiO_2_ through the LUMO level to the conduction bands [118,119], but generally have low structural stability and long-time performances. Hence, among the different organic units, the choice of polymers could generally guarantee increased stability. In particular, the organic-inorganic hybrid system that couples TiO_2_ to graphitic carbon nitride (g-C_3_N_4_) represents an intriguing solution because it meshes the photocatalytic properties of TiO_2_ with the lower band gap energy (2.7 eV) and thermal and chemical stability of g-C_3_N_4_ [120,121]. Two-dimensional conjugated polymers have emerged as a promising class of materials for photocatalytic applications thanks to their photo-physical properties such as: being eco-friendly, consisting of heart-abundant elements, easiness of synthesis, and chemically stable. Additionally, the delocalized conjugated structure of g-C_3_N_4_ gives rise to a slow charge recombination rate and a rapid photoinduced charge separation. The formation of a charge-transfer complex at the interface between the organic donor (i.e., g-C_3_N_4_) and inorganic acceptor (i.e., TiO_2_) compound results in a g-C_3_N_4_/TiO_2_ heterojunction system that shows a decrease in the recombination rate of photogenerated electron-hole pairs, as well as an increase in the photocatalytic activity of TiO_2_ under visible light for wavelengths as low as 450 nm [122,123,124,125]. The band scheme of the band structure of TiO_2_/g-C_3_N_4_ can be seen as an efficient Z-scheme where the internal electric field directing from g-C_3_N_4_ to TiO_2_ was formed because of their different work functions.

Rodrigues et al. reported a photocatalytic activity under visible radiation of the TiO_2_/g-C_3_N_4_ improved by 44.8% compared to pure TiO_2_, whereas an improvement of 30.5% was obtained under simulated solar radiation, generated by the creation of an effective Z-scheme heterojunction [126]. Further, Liu et al. develop a supramolecular self-assembly approach combined with thermal polycondensation to construct direct Z-scheme-dictated TiO_2_/g-C_3_N_4_ heterojunctions. The hybrid porous nanostructures possess hydrogen-bonded interfacial contact between TiO_2_ and g-C_3_N_4_ with enhanced visible-light photocatalytic activity in the degradation of phenolic pollutants, acetaminophen, and methylparaben, in comparison to the pure TiO_2_ and g-C_3_N_4_ [127].

The heterostructures WO_3_/g-C_3_N_4_ represent the application of an S-scheme, useful for hydrogen production [128]. In this scheme, similar to the previous example, the work function of WO_3_ (6.23 eV) is larger than that of g-C_3_N_4_ (4.18 eV), inducing the formation of an internal electric field from the polymer to the inorganic semiconductor and band-edge bending due to electron redistribution. Upon excitation, the photogenerated holes in the valence band of g-C_3_N_4_ recombine with the electrons in the conduction band of WO_3_, leaving the photogenerated carriers (electron of g-C_3_N_4_ and holes of WO_3_) free to participate in photocatalytic reactions. Following a similar scheme, CeO_2_ quantum dots (QDs) intimately anchored on a g-C_3_N_4_ have been successfully utilized for a heterostructure with efficient photocatalytic performance for bacteria disinfection [129].

ZnS nanoparticles (10–15 nm) hybridized with g-C_3_N_4_ showed photocatalytic hydrogen evolution using glucose as the substrate. Interestingly, different efficiency has been observed depending on the synthesis method of Carbon nitride (melamine, urea, and dicyandiamide). The best yield was obtained from g-C_3_N_4_ produced from melamine [130].

A further application of g-C_3_N_4_ in the hybrid systems is provided by g-C_3_N_4_/Ag_2_CO_3_ heterostructures. The photocatalytic experiments indicated that the g-C_3_N_4_/Ag_2_CO_3_ photocatalyst exhibited significantly enhanced photocatalytic activity than the pure g-C_3_N_4_ and Ag_2_CO_3_ samples toward degrading methyl orange (MO) and catalytic ozonation for oxalic acid mineralization, under visible light irradiation (λ > 420nm) [131,132].

The ZnO/K-CN hybrid exhibits excellent photocatalytic degradation and adsorption efficiency of tetracycline under simulated sunlight, and the degradation rate reaches 90% due to the synergetic action of the formation of efficient type-II heterojunction between the K-doped exfoliated g-C_3_N_4_ nanosheet (K-CN) and the ZnO nanorod and the adsorption capacity of tetracycline through electrostatic force [133].

The ZnO/g-C3N4 hybrid system was successfully utilized as a sensor with high selectivity to dimethylamine (DMA), fast response time, and a fast recovery time. The heterojunction is effective for electron transfer and adsorption of oxygen molecules forming reactive oxygen species and thereby promoting the reaction between oxygen and DMA [134].

The use of metal complexes and metal nanoparticles (Au, Pt, Ag, etc.) in exfoliated carbon nitrides was utilized in the recent year for the successful application of photocatalysis. The nanosized metal particle will provide an extra absorption band related to localized surface plasmon resonance in the visible region and the possibility of injecting hot carriers into the reaction. Ag/C_3_N_4_ photocatalysts exhibited excellent stability and enhanced visible-light-driven photocatalytic performance both in the degradation of methyl orange (MO) and H_2_ evolution from water splitting [135]. Wang et al. found that under visible light illumination, the Au/g-C_3_N_4_ hybrids catalyst exhibits a superior oxygen evolution reaction performance in alkaline electrolytes. The high activity was attributed to three factors: the hot electrons injection of localized surface plasmon effect induced by light illumination and the formation of Schottky heterostructure [136]. The formation of heterojunctions between AgCl/ZnO and g-C_3_N_4_ significantly increases electron-hole transfer and separation compared to pure ZnO and g-C_3_N_4_. Thus, AgCl/ZnO/g-C_3_N_4_ exhibits increased photocatalytic activity in the removal of tetracycline hydrochloride, reaching 90 % under visible light irradiation. Hydrogen peroxide (H_2_O_2_) and superoxide radical (·O_2_) contributed more than holes (h+) and hydroxyl radicals (·OH) to the degradation process [134]. Zhang et al. observed that, contrary to pure g-C_3_N_4_, direct splitting of pure water is achieved with graphitic carbon nitride modified with Pt, PtOx, and CoOx as redox cocatalysts [137]. The photoactivity of Au/g-C_3_N_4_ nanocomposites showed higher efficiency in the degradation of both methylene blue dye and the gemifloxacin mesylate compared with that of bare g-C_3_N_4_. The efficiency was evaluated as a function of relative concentration between gold and g-C_3_N_4_, indicating the 1%Au/g-C_3_N_4_ nanocomposites as the best among the samples studied. Under visible excitation, it was able to reach more than 95% degradation of the target dye molecule in 90 min, in contrast to less than 70% obtained by pure g-C_3_N_4_ [138].

The structure of the hybrid photocatalysts can be arranged in more articulated paths, like a plasmonic ternary hybrid photocatalyst of Ag/AgBr/g-C_3_N_4_, synthesized and used for water splitting under visible light irradiation. Compared to a simple mixture of Ag/AgBr and g-C_3_N_4_, the ternary heterostructure showed a higher photoactivity. The performances are increased thanks to different factors: the decreasing of the recombination rate into the polymeric structure due to the high separation of the photoexcited charge, the low dimension of the Ag/AgBr nanoparticles, and the enhanced absorption related to the plasmonic resonance in the visible region [139]. Further, an efficient Ag/Ag_2_WO_4_/g-C_3_N_4_ ternary plasmonic photocatalyst was successfully synthesized using a facile one-step in situ hydrothermal methods. The strong coupling effect between the Ag/Ag_2_WO_4_ nanoparticles and the exfoliated g-C_3_N_4_ nanosheets gives excellent photocatalytic results (Rhodamine B and tetracycline degradation) under visible-light excitation thanks to the synergic action of the reduced the recombination rate of photogenerated electrons and holes and plasmonic absorption [140].

The metal can play the role of a cocatalyst, too, lowering the activation energy and increasing the rate of hydrogen production. Metals such as Pt, Au, Ag, Rh, and Ni are widely used as a cocatalyst to increase the efficiency of C_3_N_4_ for hydrogen evolution [141].

In Pt/g-C_3_N_4_-GO photocatalysts, the H_2_ evolution activity is strongly influenced by the temperature of the reduction stage, so the maximal rate of H_2_ evolution was obtained for the catalyst reduced at 400, and Pt concentration of 0.5%. The quantum efficiency was about 3% [142].

The photoactivity of Au/g-C_3_N_4_ nanocomposites has been evaluated for the degradation of both methylene blue dye and the drug Gemifloxacin mesylate, and their efficiencies were compared with that of bare g-C_3_N_4_. The efficiency increased by over 95% destruction of the target dye molecule in 90 min, in contrast to the 69% achieved with bare g-C_3_N_4_ [138].

Surface plasmon resonance plays a fundamental role in the visible photocatalytic property and photoinduced electron-hole pair separation efficiency. Ag-decorated g-C_3_N_4_ reports high photocatalytic degradation efficiency of methyl orange (98.7% within 2 h) and the high catalytic reduction property of 4-nitrophenol (100% within 70 s) [143].

Au/g-C_3_N_4_/NiFe_2_O_4_ exhibited a significant visible-light-driven photoactivity for hydrogen production. The high photocatalytic performances of the ternary hybrid nanocomposites are related to the high visible absorbance for charge carrier production, the fast separation of the electrons captured by AuNPs, and the strong absorption at the localized surface plasmon band in the Au nanoparticles that generate high-density carriers under visible light irradiation [144].

A new class of hybrid photocatalysts is constituted by metal-organic frameworks (MOF), generally constituted by porous crystalline materials with metallic nodes (metal ions or clusters) and organic linkers. MOFs have recently attracted increasing attention in the field of photocatalysis for their ultra-high specific surface area (over 6000 m^2^g^−1^), rich topology, and easily tunable porous structure [145].

In some MOFs, the metal clusters and organic linkers can be regarded as isolated semiconductor quantum dots, while the organic framer act as a light-absorbing antenna [146].

Structures such as MOF-5 (Zn_4_O(BDC)_3_, BDC: 1,4-benzenedicarboxylate), (Zr_6_O_4_(OH)_4_(BDC)_6_), and Ti_8_O_8_(OH)_4_(BDC)_6_, displayed semiconductor-like behavior. Herein, the metal-oxo clusters and organic linkers can be regarded as isolated semiconductor quantum dots and light-absorbing antennae, respectively.

In MOF structures, the HOMO/LUMO levels play similar roles as the conduction and valence band in classical semiconductors. The photogenerated electrons in the LUMO can be transferred to O_2_, leading to the formation of superoxide radicals (O_2_˙^−^). Meanwhile, holes in the HOMO can oxidize the surface hydroxyl group/water, generating hydroxyl radicals (HO˙). The band gap of MOF can be modeled in a very wide range, from 3 to 4 eV MOF-5, UiO-66(Zr), and MIL-125(Ti) to a band gap lower than 2 eV in Fe- based MOF. In order to increase the photocatalytic efficiency in the visible region, different strategies have been proposed, mainly devoted to the generation, separation, and transfer of charge carriers [147]. The most studied technique to tune the optical properties is the modification of organic linkers rather than metallic modes. As an example, the use of –NH_2_ groups in H_2_BDC decreases the absorption edge down to 2.45 eV, whereas in pure H_2_BDC, the band gap is 3.6 eV, thereby extending the efficiency in the visible part of the spectrum [148].

Remarkably, the introduction of the –NH_2_ group leads to increased energy of the HOMO level with no influence on the LUMO. Similar effects have been studied for other functional groups where the –(NH_2_)_2_ group is the most active one: –CH_3_/–Cl < –OH < –(NH_2_)_2_ [149]. Similarly, it is possible to use the Covalent organic framework (COF) to obtain efficient visible photocatalysis. In MOF/COF heterostructured photocatalyst (Cu-NH_2_-MIL-125/TpPa-2-COF), with Cu ions immobilized by -NH_2_ groups of MOF, while the covalently connected heterojunction facilitates the separation of photogenerated charges. The results show an efficient “amines to imines” process with a high conversion rate (91.2%) and selectivity (>99%) [150].

In addition to functional group modification, the implantation of transition metal ions to complexes with ligands was reported to be a feasible way to enhance photocatalytic performance [151]. The implantation of Fe^3+^ in porphyrinic MOFs (PCN-224) generates an extension of the optical response to a longer wavelength inhibiting the recombination of electron-hole pairs. Lou et al. report sheet-like sliver vanadate/Bi–Fe MOF (AGV/HBF) heterojunction composite with superior efficiency under visible light irradiation (almost 99% in 2 h for the photocatalytic decomposition of Rhodamine B). The enhanced photocatalytic performance was attributed to the strong visible-light absorption and low recombination of electron-hole pairs due to the as-formed heterojunctions [152].

The dye-sensitizing approach is applied even to the MOF structure. The high specific area and the high number of bonding sites can facilitate a strong interaction and an efficient antenna effect of the dyes. The dye/MOF structure has been applied to H_2_ production and CO_2_ reduction, and environmental remediation [152].

Li et al. report porous copper-based metal–organic framework (Cu-MOF) tailored by encapsulating visible-light photosensitive dyes. The study underlying the high-efficiency and diversified performances of the different systems in the photodegradation process reveals significant achievements in tailoring the photocatalytic capabilities via introducing dyes with different photosensitivity into an identical porous copper-organic framework [153].

The coupling of MOFs with other photoactive semiconductors represents an additional strategy to enhance photocatalytic activity. Again, the high specific area of the MOF structure facilitates this approach. Three types of heterojunctions for semiconductors can be identified, depending on the relative position of the Conduction and Valence band as well as the n/p characters of the independent components, namely straddling gap; type-II: staggered gap; type-III: broken gap [101,154]. Until now, many semiconductors have been reported to form composites with MOFs. Metal-containing semiconductors (such as ZnO [155], TiO_2_ [156], BiVO_4_ [157], and CdS [158]) and non-metal graphitic carbon nitride [159] have been reported to form composites with MOFs exhibiting highly potential performance in the field of photocatalysis.

Doustkhah et al. utilized the highly porous structure of the MOF (MOF-5) as a precursor to obtaining superior photocatalytic activity in degrading methylene blue (MB) in comparison to other ZnO nanostructures [155]. Similarly, CdS/Cd-MOF nanocomposites were constructed by in situ sulfurizations to form CdS using Cd-MOF as a precursor. The role of the high specific surface is evidenced by the high degradation rate of MB (91.9%) in comparison with the pure Cd-MOF (62.3%) and pure CdS (67.5%) [158].

## 6. Organic/Inorganic Heterostructures: Applications

Organic-Inorganic hybrids have been widely studied as efficient photocatalysts, degradation of organic pollutants, hydrogen evolution, CO_2_ reduction, sterilization, and reduction of heavy metals are the most studied. In Table 1 are collected some examples of hybrid photocatalysts for each application.

### 6.1. Environmental Applications: Pollutants Degradation, Reduction of Heavy Metals, Sterilization

**Figure 11:**
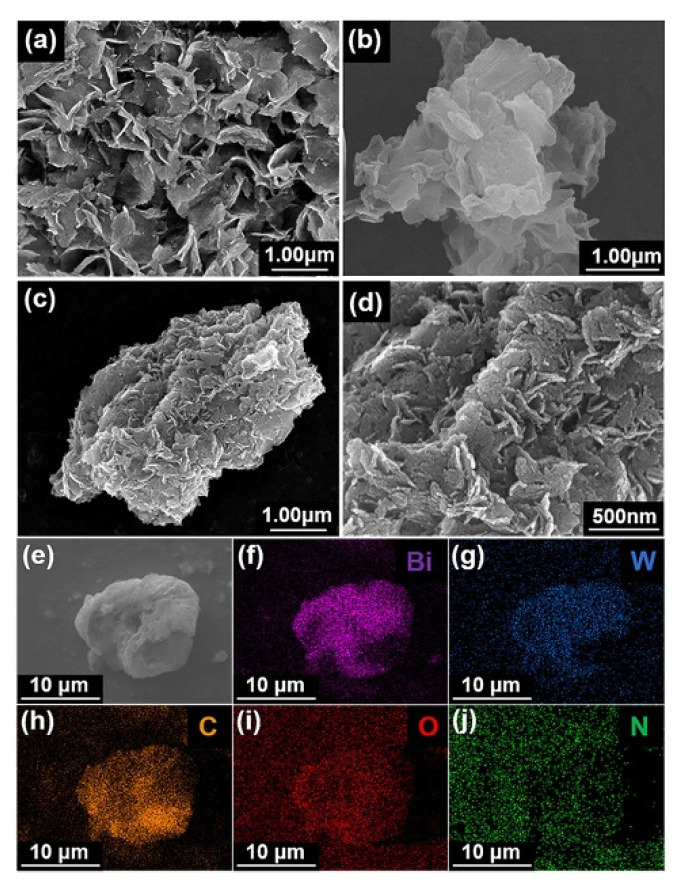
SEM images of (**a**) BWO nanosheets, (**b**) PI, (**c**,**d**) 30% PI@BWO and magnification, (**e**) FE-SEM images of 30% PI@BWO and (**f**−**j**) EDX element mapping images of Bi, W, C, O, and N.

**Figure 12:**
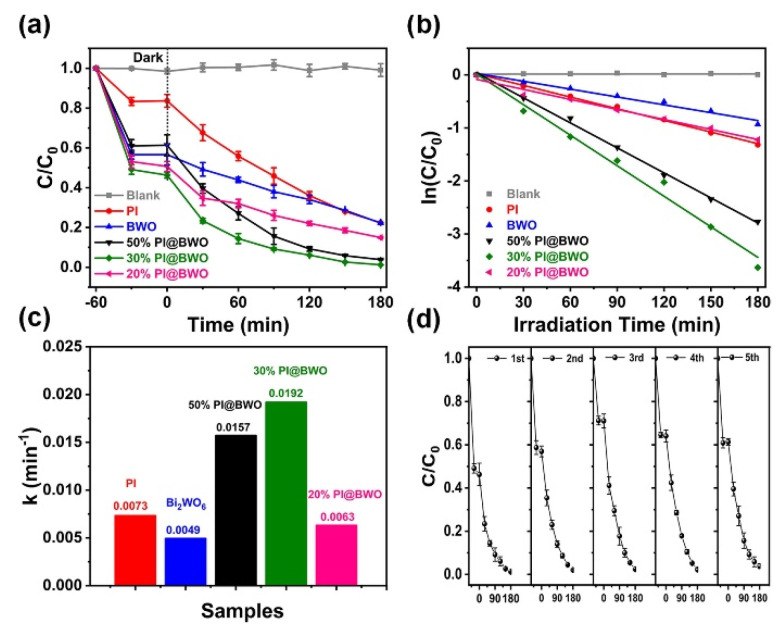
(**a**) BPA photocatalytic removal performance over as-prepared samples under visible light illumination, (**b**) pseudo-first-order kinetics equation fitted with the BPA degradation processes over different samples, (**c**) kinetic rate constants (min^−1^) of the samples, (**d**) five consecutive photocatalytic degradation cycles over 30% PI@BWO [161].

In the field of the degradation of pollutants, Han et al. [161] describe how the π-π stacking in perylene imide/Bi_2_WO_6_ hybrid photocatalyst enhances the photocatalytic activity for the degradation of Bisphenol A (BPA), also underlining the good stability and recyclability. The 2D sheet-like structure observed in the SEM measurements promotes the presence of a high concentration of active sites on the surface. The presence of the polymer facilitates the separation of the charge carriers, increasing the photocatalytic activity joined to the nanosheet-like structure that increases the number of active sites for the reaction (Figure 11). The photocatalytic tests show that by increasing the percentage of Bi2WO3 in the hybris first, the photocatalytic activity increases and then decreases, and the best activity has been observed when 30% of Bi_2_WO_3_ is present, and it is stable even after five cycles (Figure 12).

**Figure 13:**
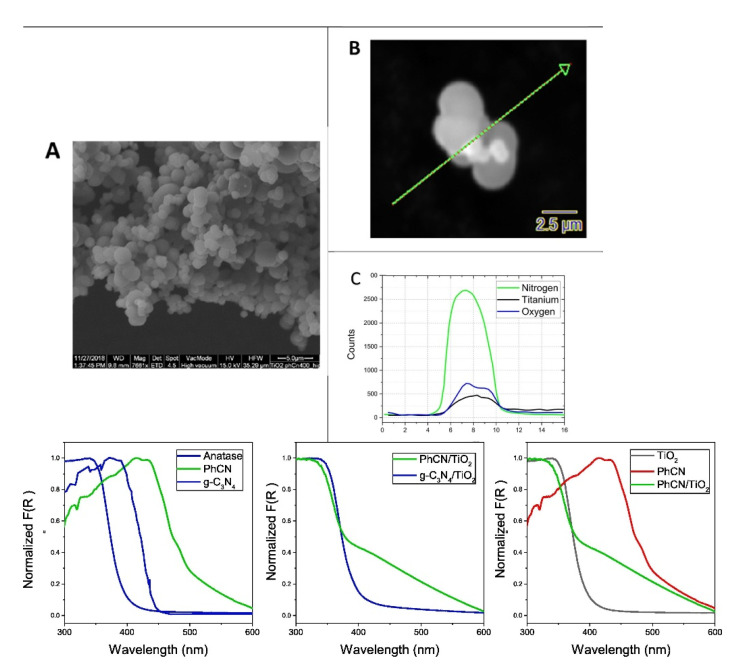
SEM images of PhCN/TiO_2_ hybrid system taken at low (**A**) and high magnification (**B**) and EDX analysis for the cross-section denoted by the dashed green. Normalized absorption spectra of TiO_2_ (anatase), g-C_3_N_4_, and PhCN (i.e., the building blocks for the hybrid systems) (**A**), the hybrid systems g-C_3_N_4_/TiO_2_ and PhCN/TiO_2_ (**B**), and the differential absorption of PhCN/TiO_2_ from building blocks (**C**) [166].

The modifications through the modulation of the organic mesh represent a further strategy to increase the photocatalytic activity of the material. The introduction of phenyl rings in the mesh of g-C_3_N_4_ was utilized to shift the absorption edge in the visible range and to obtain an efficient hybrid photocatalyst (Phenyl modified-TiO_2_ hereafter PhCN) up to 600 nm (Figure 13) [96,166]. The absorption spectrum of the hybrid system appears as the sum of the two building blocks.

**Figure 14:**
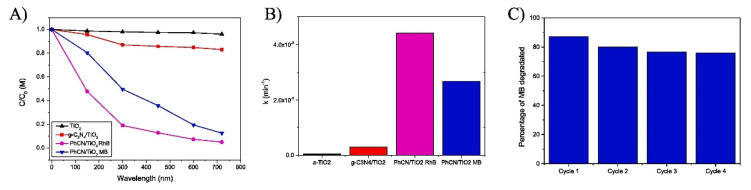
PhCN/TiO_2_ photocatalyst and its application for the degradation of RhB and MB (**A**). Rate constant (k) by employing TiO_2_ (anatase), g−C_3_N_4_/TiO_2_, and PhCN/TiO_2_ as photocatalysts respectively (**B**). The stability of the catalyst is reported for the MB photodegradation (**C**) [166].

The phenyl-modified carbon nitride presents lower band gap LUMO levels almost resonant with the conduction band of TiO_2_; the electrons are promoted from the HOMO ground state into the LUMO excited state of the phenyl carbon nitride and then transferred to the conduction band of the TiO2. The degradation efficiency for Rhodamine B and Methylene Blue under white LED irradiation increased from 4% by using pure compounds to 17% by using only g-C_3_N_4_/TiO_2,_ raising to 98% and 88% with the hybrid system PhCN/TiO_2_ (Figure 14).

**Figure 15:**
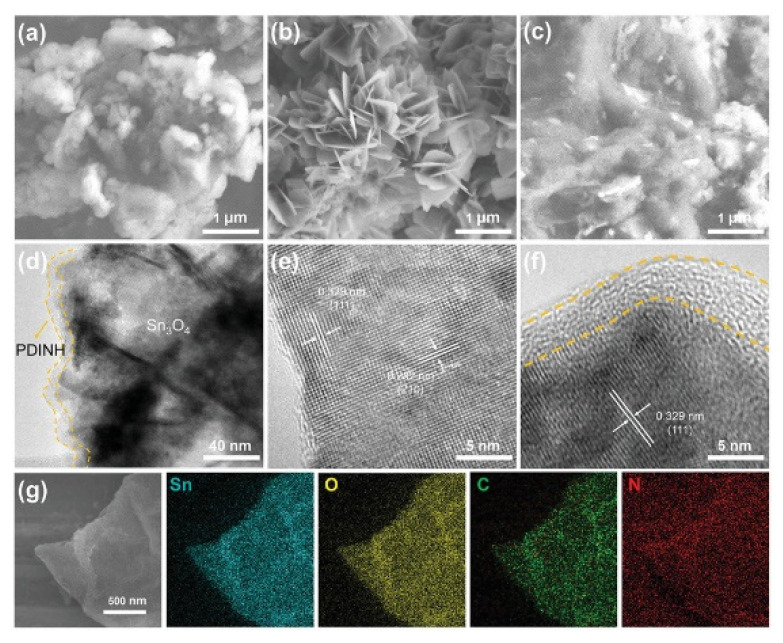
SEM images of (**a**) PDINH, (**b**) Sn_3_O_4_, and (**c**) Sn_3_O_4_/PDINH−10%. (**d**) TEM image of Sn_3_O_4_/PDINH−10%. HRTEM images of (**e**) Sn_3_O_4_ and (**f**) Sn_3_O_4_ /PDINH-10%. (**g**) EDS element mapping of Sn_3_O_4_/PDINH−10%.

**Figure 16:**
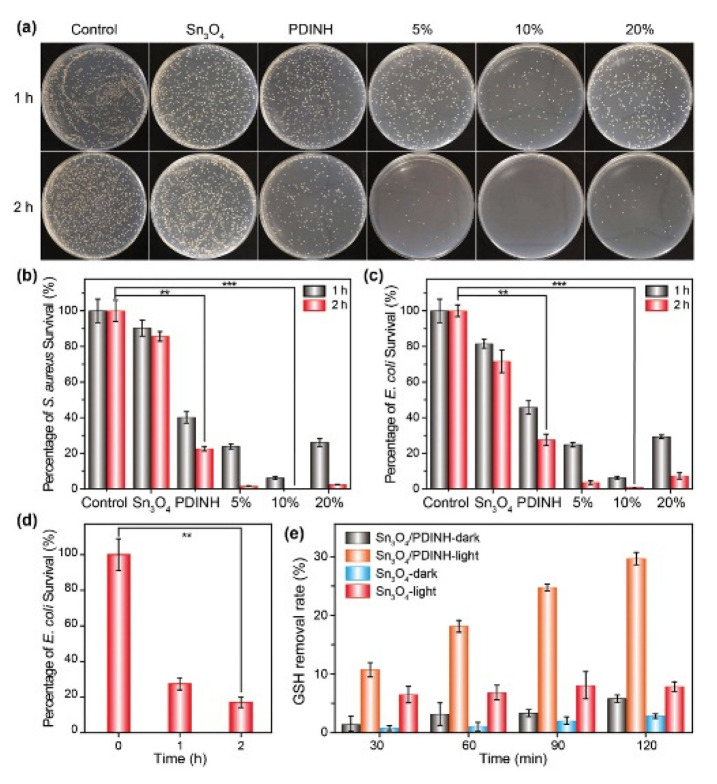
Antibacterial activity. (**a**) Photographs of antibacterial results under simulated sunlight illumination (*S. aureus*). Antibacterial activities of the different materials under simulated sunlight illumination: (**b**) *S. aureus* and (**c**) *E. coli*. (**d**) Antibacterial performance of Sn_3_O_4_/PDINH heterostructure under NIR irradiation (λ > 700 nm). (**e**) GSH losing histograms after incubation with materials under simulated sunlight illumination or not [180].

New methods for killing antibiotic-resistant bacteria have gained large interest in the last year, and in this field, photocatalysis is widely used. The hybrid photocatalyst studied by Yang et al. [180] appears to be very efficient in capturing bacteria. TEM images of the Sn_3_O_4_/PDINH hybrid show an organic amorphous layer with a thickness of about 5nm on the surface of Sn_3_O_4_ nanosheets. The formation of the composite has been further confirmed by EDS analysis, which reveals the presence of Sn, O, C, and N (Figure 15). This hybrid photocatalyst obtained by coupling Sn_3_O_4_ and perylene diimide, thanks to the π-π bonding interactions, allows generating active oxygen species capable of killing the bacteria because these species interact with proteins, lipids, and other components of the bacteria, causing death. The photocatalytic antibacterial activity has been evaluated by performing the plate-counting method. The highest observed capture rate for this hybrid has been 82%, improved by the presence of the organic polymer on the surface (Figure 16).

**Figure 17:**
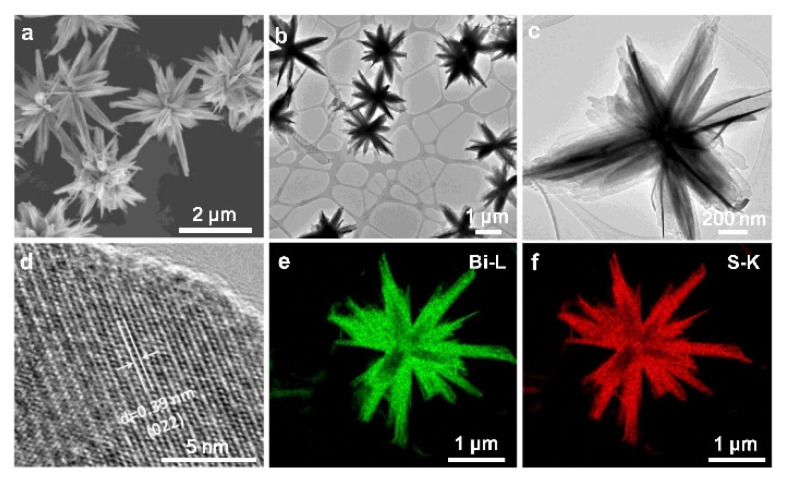
(**a**) SEM and (**b**,**c**) TEM images with different magnifications of Bi_2_S_3_−1. (**d**) HRTEM image and (**e**,**f**) EDS elemental mapping of Bi_2_S_3_−1.

**Figure 18:**
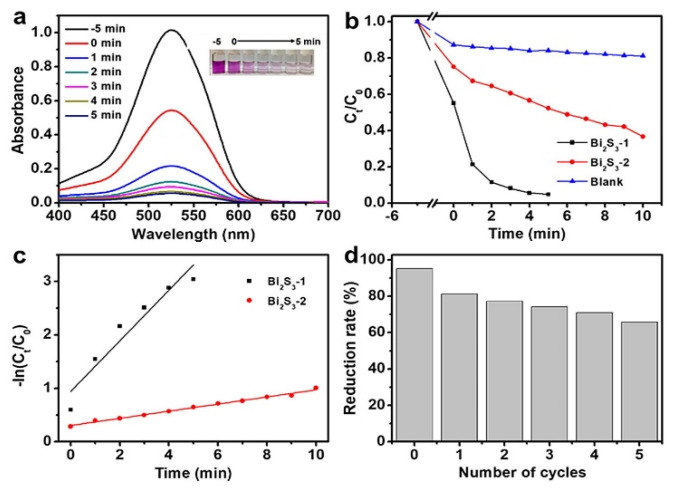
(**a**) Absorption spectra of RhB solution at different times of photodegradation with Bi_2_S_3_-1 (the inset in the right corner is an image of RhB solution before and after photodegradation). (**b**) Variation of RhB concentration with photodegradation time in the existence of various photocatalysts (Bi_2_S_3_−1, Bi_2_S_3_−2, and TiO_2_) under visible light. (**c**) −ln (Ct/C0) versus time curves of Bi_2_S_3_−1, Bi_2_S_3_−2 and TiO_2_. (**d**) The durability tests of Bi_2_S_3_−1 for photodegradation of RhB [182].

Shen et al. synthesized novel three-dimensional Bi_2_S_3_ nanocrystals capped by polyvinyl pyrrolidone photocatalyst for the reduction of hexavalent chromium under visible light irradiation. TEM images display flower-like structures consisting of nanosheets (Figure 17). The reduction of metals with such an impressive photocatalyst reaches 95% in only 5 min of reaction (Figure 18).

### 6.2. Energy: CO_2_ Reduction and H_2_ Evolution

The formation of two-dimensional (2D) nanostructures of the polymeric structure is a successful strategy to increase the photocatalytic activity: higher aspect ratio, larger surface area, and increased number of surface groups for anchoring cocatalysts are fundamental properties of the layered structures. Two-dimensional g-C_3_N_4_ nanosheets have been successfully obtained, and significantly photocatalytic performance was reported [97,133].

**Figure 19:**
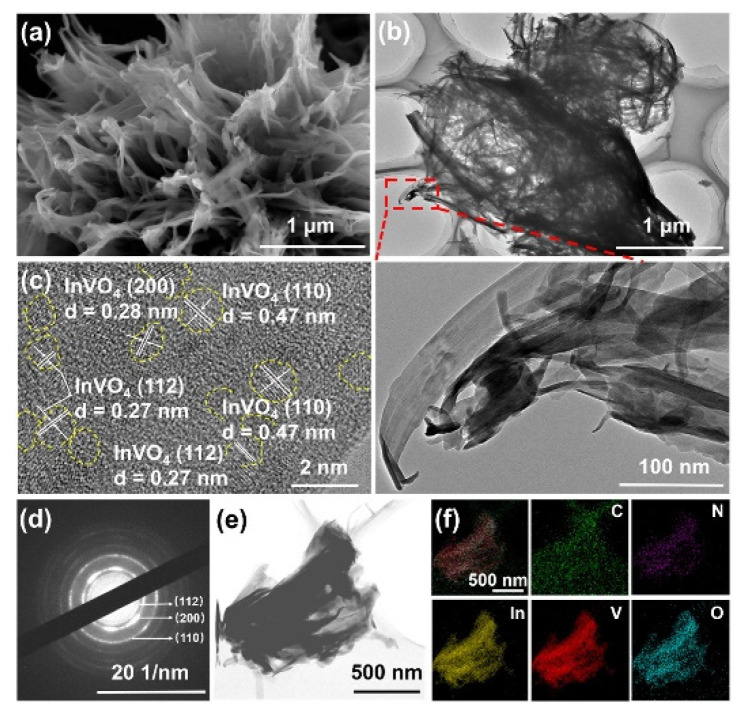
SEM image (**a**), TEM images (**b**), HRTEM image (**c**), SAED pattern (**d**), HAADF−STEM image (**e**) and corresponding EDX elemental mapping images (**f**) of InVO_4_/CN−II.

**Figure 20:**
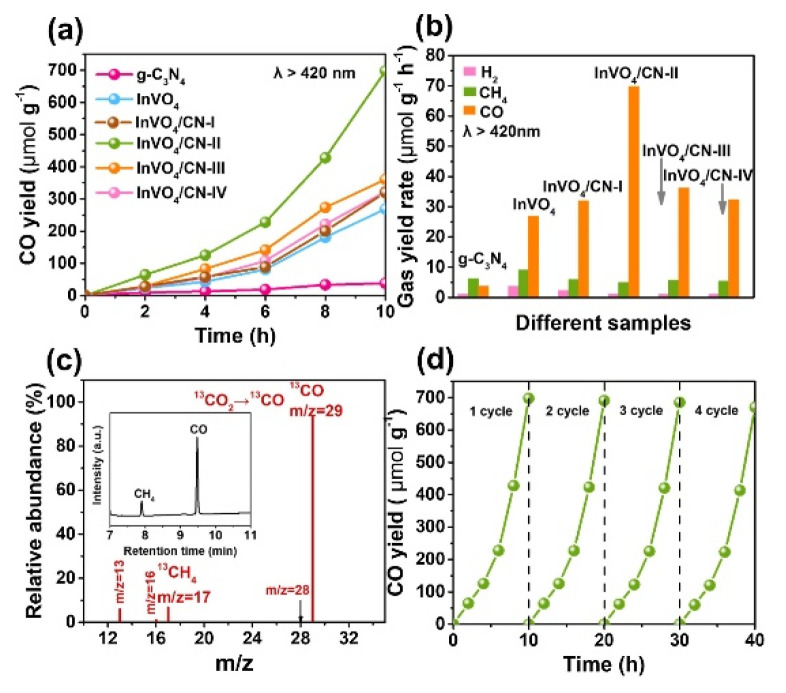
(**a**) CO generation and (**b**) average gas evolution rates of H_2_, CH_4_, and CO in MeCN/H_2_O system over various photocatalysts for 10 h photocatalytic reaction under visible light irradiation (with a UVCUT−420 nm filter). (**c**) Mass spectrum of 13CO (m/z = 29) produced over InVO_4_/CN-II in the photoreduction of 13CO_2_. (**d**) Cycling production of CO over InVO_4_/CN−II under visible light irradiation [173].

For CO_2_ reduction, a new and very stable 0D/2D hybrid system has been proposed by Gong et al. [173]. This photocatalyst, with a high surface area, presents high photocatalytic activity because of the presence of a large number of active sites. In SEM images, the hybrids systems show the nanosheet structure and no adhesive nanoparticles on the surface (Figure 19). The observed reduction rate for the CO_2_ reduction has been 69.8 μmol g^−1^g^−1^ under visible light irradiation (Figure 20).

The production of H_2_ as an energy source has been studied increasingly in the last few years. Hamdy et al. [168] propose a novel polyaniline/ZnO heterojunction, synthesized via sol-gel/polymerization route for photocatalytic hydrogen production under a visible light source.

**Figure 21:**
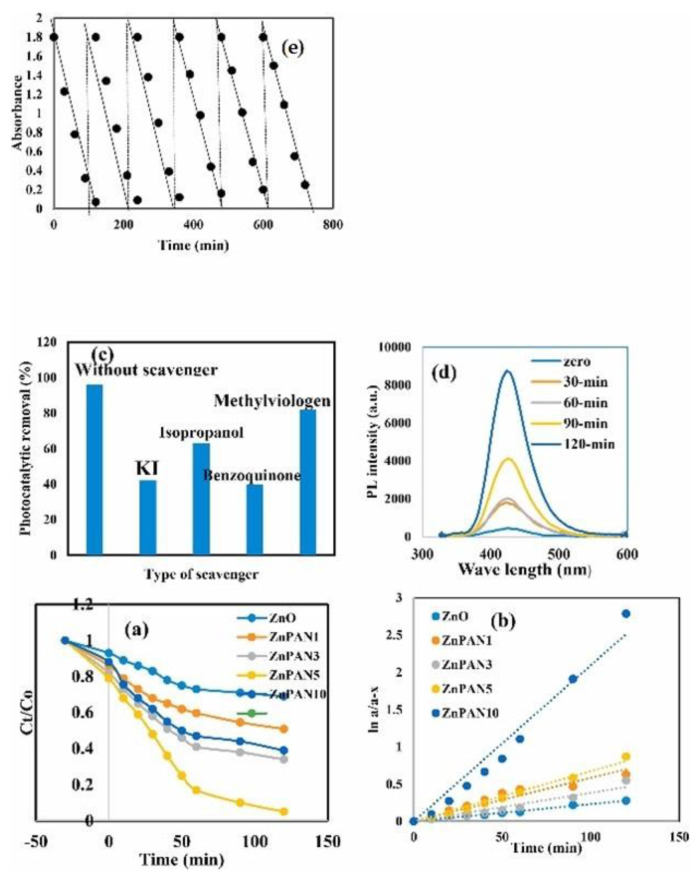
(**a**) Photocatalytic degradation of FLU dye over ZnO, ZnPAN1, ZnPAN3, ZnPAN5, and ZnPAN10, (**b**) Pseudo-first order kinetic for photodegradation of FLU dye over ZnO, ZnPAN1, ZnPAN3, ZnPAN5 and ZnPAN10, (**c**) The effect of scavengers on photocatalytic degradation of FLU dye over ZnPAN5, (**d**) PL spectrum for terephthalic acid on the sample ZnPAN5 (**e**) Re-cyclic of ZnPAN5 for five consecutive cycles [168].

The amount of polymer has a fundamental role in photocatalytic activity as it plays a uniform deposition of it on the ZnO surface (Figure 21).

**Figure 22:**
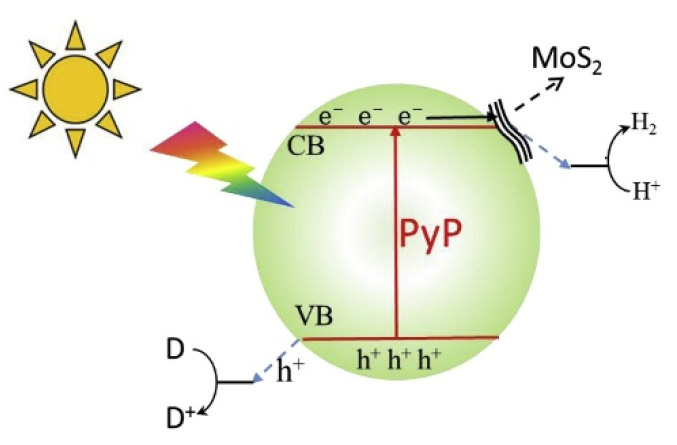
Schematic illustration of the between PyP and MoS_2_. D = donor [170].

Zang et al. showed how the p-conjugation, the flexibility, and the high surface area of pyrene-based polymer coupled with MoS_2_ enhance photocatalytic H_2_ production [170]. Under visible light irradiation, the electrons can be quickly transferred from the conduction band of pyrene-based polymer to MoS_2_ restraining the charge recombination process (Figure 22).

**Figure 23:**
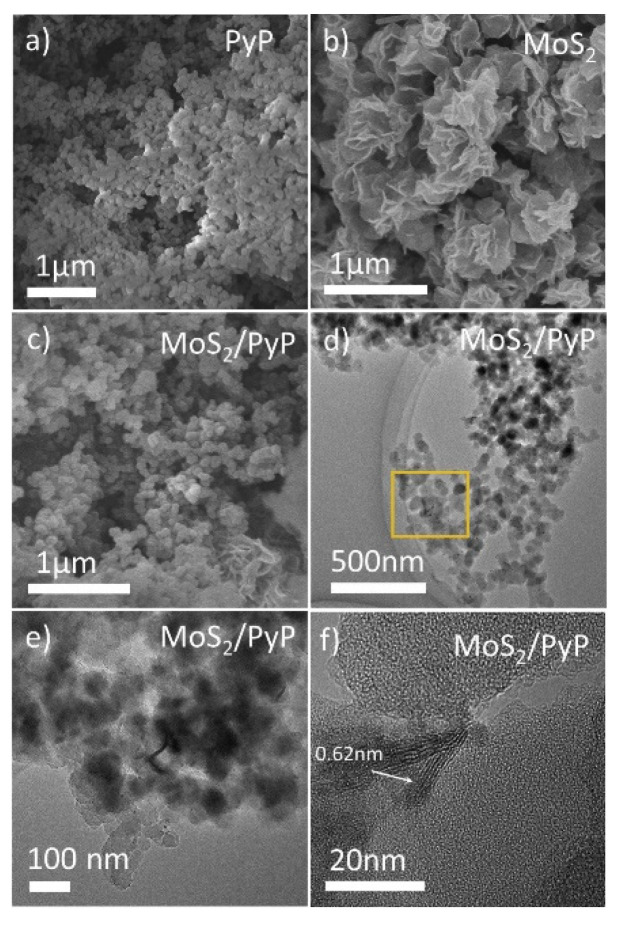
SEM images of (**a**) pure PyP, (**b**) MoS2 (**c**) 3 wt. % MoS2/PyP(IM) samples; (**d**–**f**) TEM images of 3 wt. % MoS_2_/PyP(IM) samples.

From SEM and TEM measurements, it is possible to observe the different morphology of the two components of the hybrid system. The PyP particle size is 40–100 nm while MoS_2_ shows a nanosheet-like structure with few nanometers of thickness, as it is well distinguishable in the TEM images (Figure 23). The photocatalytic activity is enhanced by the fast transfer of the excited electrons from the conduction band of the PyP to the MoS_2,_ whose surface is covered with active sites, allowing the photocatalytic process.

**Figure 24:**
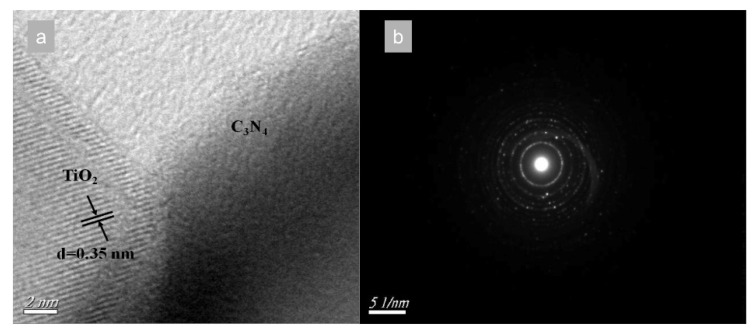
The HR−TEM image (**a**) and SAED pattern (**b**) of C_3_N_4_/TiO_2_ composite photocatalysts [177].

Wang et al. designed TiO_2_-modified g-C_3_N_4_ composite photocatalysts using the ball milling synthesis method followed by a calcination process. From spectroscopic measurements and morphological studies, they confirmed the formation of the heterostructure that reflects an enhancement of the separation rate of the photogenerated electrons and holes and, therefore, of the CO_2_ reduction rate (Figure 24) [177].

**Figure 25:**
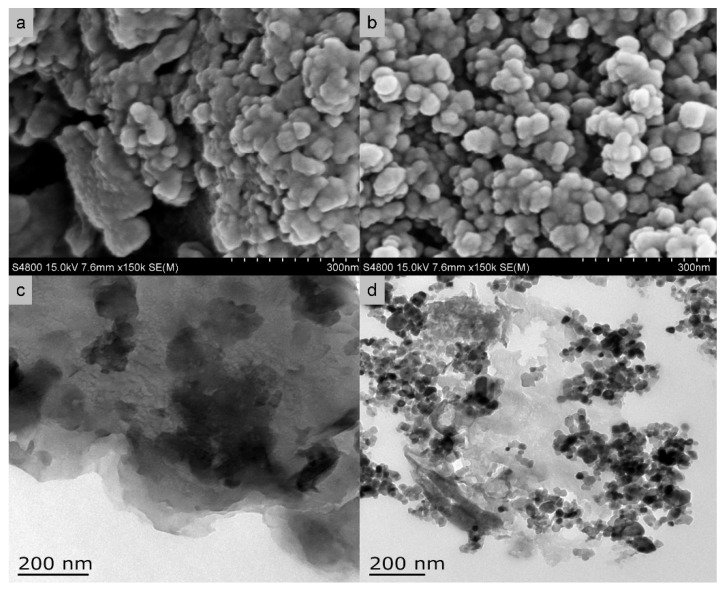
SEM (**a**,**b**) and TEM (**c**,**d**) images of as prepared photocatalysts: (**a**,**c**) pure bulk C_3_N_4_, (**b**,**d**) C_3_N_4_/TiO_2_ composite photocatalysts.

From SEM analysis emerges an irregular structure with aggregates of bulk g-C3N4 and TiO_2_ nanoparticles with 20–50 nm size on its multilayered surface (Figure 25). The formation of the heterostructure promotes the separation between the electron and the holes, and the best photocatalytic result shows the yields of 72.2 and 56.2 μmol g^−1^ for CH_4_ and CO, respectively

In summary, hybrid organic/inorganic photocatalysts have emerged as highly potential materials to drastically improve visible photocatalytic efficiency. Our review cannot be fully comprehensive for the large amount of new scientific products that are available daily, but it would represent a guide for readers that want to enter more insights into the field of photocatalysis. More specifically, we presented the high potential impact of the use of visible light for environmental remediation and energy production.

## 7. Conclusions and Outlook

“Set the controls for the heart of the sun”, sang Pink Floyd; we can now better paraphrase the title as “Set the control for the use of the heart of the sun”. The visible-driven photocatalytic process has become a rapid and important strategy to mitigate the anthropogenic impact and mitigate energy consumption. Even if different solutions have already been proposed and, in some cases, good performances have been achieved, the road is still long. Starting from the wide galaxy of the actual literature, it will be possible to set the control of the best parameters to use solar radiation in the photocatalytic properties.

In this review, we analyzed the state art inorganic and organic semiconductors underlining how the improvement of the absorption in the visible range represents a crucial point in the actual research and how the study of heterostructures can overcome this problem.

New Materials with reduced environmental impact, the use of hybrid solutions to overcome the limitations of pure inorganic and organic materials, surface modification, doping processes, and new heterostructures are important parameters to set the more appropriate energy level and recombination process.

The review aims to provide a useful guideline for designing and developing new photocatalysts useful to find suitable roads to achieve the use of the Heart of the Sun.

## Figures and Tables

**Table 1:** Organic-Inorganic photocatalysts and applications.

Application	Photocatalyst	Photocatalysts Target	Source of Light	Efficiency	Ref.
Degradation of organic pollutants	Citric acid/CeO_2_	50 mgL^−1^ glyphosphate	UV lamp 800 W/m^2^, 365 nm and visible lamp 1800 W/m^2^, 400–800 nm	100%	[160]
	Perylene imide/Bi_2_WO_6_	50 mL Bisphenol A 10 ppm	300 W Xe lamp cold light source	100%	[161]
	Ag_3_PO_4_/PDI	20 mgL^−1^ tetracycline hydrochloride	300 W xenon lamp with a cut filter (>420 nm)	82%	[162]
	WO_3_/Cu/PDI	20 mgL^−1^ tetracycline hydrochloride	300 W Xe lamp with a cut-off filter (λ > 420 nm)	75%	[163]
	ZnSe/Polyaniline	10 mgL^−1^ tetracycline hydrochloride	LED with 50 W powers and 4300 LM as a visible-light source	90%	[164]
	g-C_3_N_4_/TiO_2_	200 mL Bisphenol A, Brilliant green and mixed dyes 20 mgL^−1^	Direct sunlight	95%	[165]
	PhCN/TiO_2_	50 mL RhB/MB 10 mgL^−1^	white LED Philips 13 W with optical power 100 mW	98%	[166]
	S-doped g-C_3_N_4_/TiO_2_	100 mL Congo Red 50 mgL^−1^	300 W Xenon lamp	>90%	[167]
Hydrogen evolution	Polyaniline/ZnO	H_2_O	Xenon lamp	9.4 mmolh^−1^g^−1^ H_2_	[168]
	Cds/TCP	H_2_O	300 W Xe-lamp UV cut-off filter λ ≥ 420 nm	104.51 mmolh^−1^g^−1^ H_2_	[169]
	Pyrene/MoS_2_	H_2_O	A 300 W Xeon lamp with a working current of 15 A	2.7 mmolh^−1^ g^−1^ H_2_	[170]
	Ti-phosphonate/MOF	H_2_O	visible-light irradiation (λ > 400 nm)	1260 mmolh^−1^g^−1^ H_2_	[171]
	gC_3_N_4_/TiO_2_/Ti_3_C_2_	H_2_O	simulated visible light (Xe 300 W lamp)	2592 μmolh^−1^ g^−1^ H_2_	[172]
CO_2_ reduction	InVO_4_/g-C_3_N_4_	CO_2_ and H_2_O	visible light irradiation (λ > 420 nm)	69.8 mmolh^−1^g^−1^ CO	[173]
	Graphydine/Bi_2_WO_6_	CO_2_ and H_2_O	simulated sunlight irradiation	2.13 mmolh^−1^g^−1^ CH_3_OH	[174]
	Zr(IV)/MOF/Co	CO_2_ and H_2_O	visible-light irradiation	70.8 mmolh^−1^g^−1^ CH_4_	[175]
	SnNb_2_O_6_/CdSe-DETA	CO_2_ and H_2_O	visible-light irradiation (λ > 400 nm)	36.16 mmolh^−1^g^−1^ CO	[176]
	g-C_3_N_4_/TiO_2_	CO_2_ and H_2_O	8 W UV-light lamp	56.2 μmolh^−1^ g^−1^ CO	[177]
Sterilization	CeO_2_/polymeric CN	*Staphylococcus aureus*	visible-light irradiation (λ ≥ 420 nm)	88% sterilization	[129]
	a-Fe_2_O_3_/g-C_3_N_4_	*Escherichia coli*	300 W Xenon lamp, 400 nm ultraviolet cut-off filter	>80% sterilization	[178]
	P-doped MoS_2_/g-C_3_N_4_	*Escherichia coli*	Visible light	100% sterilization	[179]
	Sn_3_O_4_/PTCDI	*Staphylococcus aureus* and *Escherichia coli*	Simulated sunlight	>90% sterilization	[180]
Reduction of heavy metals	TiO_2_/WO_3_/PANI	Cr(VI) 10 mgL^−1^	400 W visible lamp (2600 Lux)	68%	[181]
	PVP/Bi_2_S_3_	Cr(VI) 10 mgL^−1^	Visible light	95%	[182]
	PW_12_/CN/Bi_2_WO_6_	Cr(VI) 20 mgL^−1^	1KW xenon lamp (λ > 420 nm)	98%	[183]
